# Heterochromatin as an Important Driver of Genome Organization

**DOI:** 10.3389/fcell.2020.579137

**Published:** 2020-09-18

**Authors:** Andrés Penagos-Puig, Mayra Furlan-Magaril

**Affiliations:** Department of Molecular Genetics, Institute of Cellular Physiology, National Autonomous University of Mexico, Mexico City, Mexico

**Keywords:** heterochromatin, genome organization, phase separation, chromatin compartments, lamins

## Abstract

Heterochromatin is a constituent of eukaryotic genomes with functions spanning from gene expression silencing to constraining DNA replication and repair. Inside the nucleus, heterochromatin segregates spatially from euchromatin and is localized preferentially toward the nuclear periphery and surrounding the nucleolus. Despite being an abundant nuclear compartment, little is known about how heterochromatin regulates and participates in the mechanisms driving genome organization. Here, we review pioneer and recent evidence that explores the functional role of heterochromatin in the formation of distinct chromatin compartments and how failure of the molecular mechanisms forming heterochromatin leads to disarray of genome conformation and disease.

## Introduction

While working on cytological preparations of liverwort chromosomes, botanist Emil Heitz coined the term ‘heterochromatin’ to distinguish regions that remained strongly stained throughout the cell cycle from those that became invisible during interphase (Heitz, 1928). Due to its highly compacted state, Heitz hypothesized that heterochromatin zones were genetically inactive, laying the foundations to study the interplay between chromatin compaction and gene expression regulation (Berger, 2019).

The first link between gene silencing and heterochromatin came from observations made by H. J. Muller in the fruit fly. He identified a series of X-ray induced chromosome rearrangements that caused a variegated phenotype in the pigmentation of the fly’s eyes due to *white* gene expression inactivation, without alterations in the gene sequence (Muller, 1930; Muller and Altenburg, 1930). Schultz (1936) later demonstrated that this inactivation resulted from relocation of the gene into proximity of a heterochromatic region, suggesting that heterochromatin could influence gene activity (Schultz, 1936). Subsequent research on heterochromatin formation *de novo* during cell differentiation prompted the idea of a dynamic state of chromatin compaction that is responsive to developmental and environmental cues and the distinction between constitutive and facultative heterochromatin (Brown, 1966).

Transposable elements were the first genetic elements identified within heterochromatin that require silencing in a healthy cell (McClintock, 1951). Similarly, silenced satellite repeated sequences were mapped to the pericentromeric regions of chromosomes, providing further evidence of heterochromatin comprising a repressive compartment (Britten and Kohne, 1968; Jones, 1970).

The following decades were marked by important breakthroughs characterizing mechanisms underlying gene inactivation, mainly, DNA methylation (Holliday and Pugh, 1975; Riggs, 1975), nucleosome composition and post-translational histone modifications (Kornberg and Thomas, 1974; Brownell et al., 1996; Luger et al., 1997). In the following years and up to today, extensive molecular profiling of heterochromatin has been possible through chromatin immunoprecipitation (ChIP) technologies (Nakayama et al., 2001; Boyer et al., 2006; Li and Zhou, 2013).

In the last decade, an increasing amount of evidence has shown that the nuclear location of DNA sequences coincides with particular transcriptional states. Heterochromatin aggregates in discrete bodies inside the nucleus and at the nuclear periphery, and repositioning of a gene from the nuclear periphery toward the interior often correlates with changes in its expression levels (Kosak et al., 2002; Pickersgill et al., 2006).

Furthermore, the development of Chromosome Conformation Capture technologies, in particular Hi-C, based on the proximity-dependent ligation and sequencing of restricted DNA fragments confirmed that the genome is indeed spatially partitioned in chromatin compartments corresponding to euchromatin and heterochromatin and led to the discovery of Topological Associated Domains (TADs), which have an important role delimiting functional interactions between distant regulatory elements and genes regulating their expression (Lieberman-Aiden et al., 2009; Dixon et al., 2012; Rao et al., 2014).

However, there are still many questions on how heterochromatin can restrain or promote specific DNA interactions and contribute to the formation of distinct chromatin domains and compartments. In this review, we address stimulating current evidence that implies an active role of heterochromatin in 3D genome organization establishment and maintenance and the proposed molecular mechanisms of heterochromatin mediated structure in health and disease.

## Heterochromatin Types and Establishment

Heterochromatin is categorized into two major types, constitutive and facultative. Constitutive heterochromatin (CH) refers to condensed regions that are consistently silenced in all cell types of an organism and comprises pericentromeric and telomeric repeated sequences, transposons and some gene-poor regions of the genome. CH is molecularly defined by the presence of H3K9me3, a modification carried out by the histone methyltransferases (HMT) Suv39h in mammals, Su(var)3-9 in *Drosophila* and Clr4 in yeast (Rea et al., 2000; Peters et al., 2001). These HMTs are able to self-propagate heterochromatin since they recognize H3K9me3 and methylate adjacent nucleosomes (Al-Sady et al., 2013; Muller et al., 2016). Heterochromatin protein 1a (HP1a) in *Drosophila* and its orthologs in mammals (HP1α) and *S. pombe* (Swi6) are composed by two domains separated by a hinge region: an N-terminal chromodomain that binds H3K9me3 and a C-terminal chromo-shadow domain that serves as a platform for HP1 dimerization and binding of numerous chromatin-modifying proteins and components of the nuclear envelope thus promoting heterochromatin spreading through large domains (Kwon and Workman, 2008; Eissenberg and Elgin, 2014).

Facultative heterochromatin (FH) consists of cell-type-specific heterochromatic regions that retain their potential to switch into euchromatin under certain cues and is frequently present at developmental genes. FH is marked by the presence of the Polycomb repressive complexes 1 and 2 (PRC1 and PRC2), the latter being responsible for the deposition of H3K27me3, a histone mark associated with FH (Cao et al., 2002).

Although it is referred to as a repressive compartment, there is still low RNA synthesis in heterochromatin and RNA molecules are required to recruit the machinery necessary for heterochromatin formation. Some examples are: siRNAs processed from pericentromeric sequences in *S. pombe* and the long non-coding RNA *Xist* from the mammalian X chromosome are required for gene inactivation (Volpe et al., 2002; Chu et al., 2015). Also major satellite repeat transcripts sequester HP1α to promote heterochromatin maturation in mESC (Novo et al., 2020). Finally, piwi-interacting RNAs (piRNAs), small RNAs involved in post-transcriptional silencing of transposons in the animal’s germline, arise from clusters of repeated elements embedded in heterochromatin (Brennecke et al., 2007; Aravin et al., 2008) and their transcription is enforced by the HP1 variant Rhino dependent recruitment of transcription factors in *Drosophila* (Andersen et al., 2017).

In *S. pombe*, heterochromatin maintenance is regulated by RNA in a dosage-dependent manner as both overexpression and depletion of RNAse H disrupt heterochromatin (Nakama et al., 2012). Similarly, defects in RNA decapping and degradation in cells lacking Caf1, a member of the Cccr4-Not complex, provoke transcriptional activation of subtelomeric regions and a decrease in H3K9me2 at CH loci (Bronner et al., 2017). Depletion of components of the Cccr4-Not complex also cause derepression of transposons in *Drosophila* and *C. elegans* (Fischer et al., 2013; Morgunova et al., 2015; Kordyukova et al., 2020) and RNAseA treatment alters heterochromatin stability and localization in mice (Thakur et al., 2019). Thus, RNA-mediated regulation might be a conserved mechanism to maintain heterochromatin stability.

The mechanisms involved in heterochromatin formation, spread and maintenance are complex and act coordinately to assure gene expression silencing. The detailed molecular signals that trigger heterochromatin formation *de novo* have not been fully characterized, hence further studies are needed to define the function of new actors implicated in heterochromatin remodeling leading to better understanding of, or even control of, its formation.

## Emerging Function of Heterochromatin in Genome Topology

### Heterochromatin Positioning Within the Cell Nucleus

Electron microscopy images of the cell nucleus prompted the idea of heterochromatin forming large-scale compartments occupying distinct positions, particularly at the nuclear periphery and around the nucleolus. These genomic domains have been named Lamina-Associated Domains (LADs) and Nucleolus-Associated Domains (NADs) respectively and suggest a relationship between heterochromatin positioning and gene expression regulation.

LADs are defined as chromatin regions associated with components of the nuclear envelope that tether them to the nuclear periphery. They are rich in H3K9me2/3 and have low gene density and/or lowly expressed genes (Guelen et al., 2008). In mammalian cells, LADs are present in all chromosomes and can make up to 30% of the genome, comprising a major heterochromatin compartment (Meuleman et al., 2013).

Although the mechanisms of LAD formation remain unclear, it seems to depend on the activity of adapter proteins able to bind H3K9me2/3 and interact with components of the nuclear envelope. HP1α, for example, binds the Lamin B Receptor (LBR) and both Lamin A and B in mammals. In *C. elegans* CEC-4 protein localizes stably in the nuclear envelope and binds directly to H3K9me (Ye and Worman, 1996; Gonzalez-Sandoval et al., 2015; Gesson et al., 2016). Accordingly, elimination of the genes encoding Lamin A/C, LBR, or CEC-4 result in LAD disruption, heterochromatin mislocalization and in some cases aberrant gene transcriptional activation (Solovei et al., 2013; Gonzalez-Sandoval et al., 2015).

Heterochromatin positioning in the interphase nucleus is, to some extent, influenced by the chromosome arrangement during cell division. Centromeres and telomeres cluster on opposite sides of the nuclear periphery in plants, yeast, and *Drosophila* (Cowan et al., 2001). This distribution is termed Rabl configuration and depends on the association between the centromeres and microtubules of the cytoskeleton and seems to preserve the chromosome orientation observed in anaphase (Jin et al., 2000; Therizols et al., 2010). In mammals, the lamina-associated polypeptide LAP2α binds chromosomes during anaphase and mediates LADs re-assembly in concert with other proteins of the nuclear envelope (Samwer et al., 2017).

**FIGURE 1:**
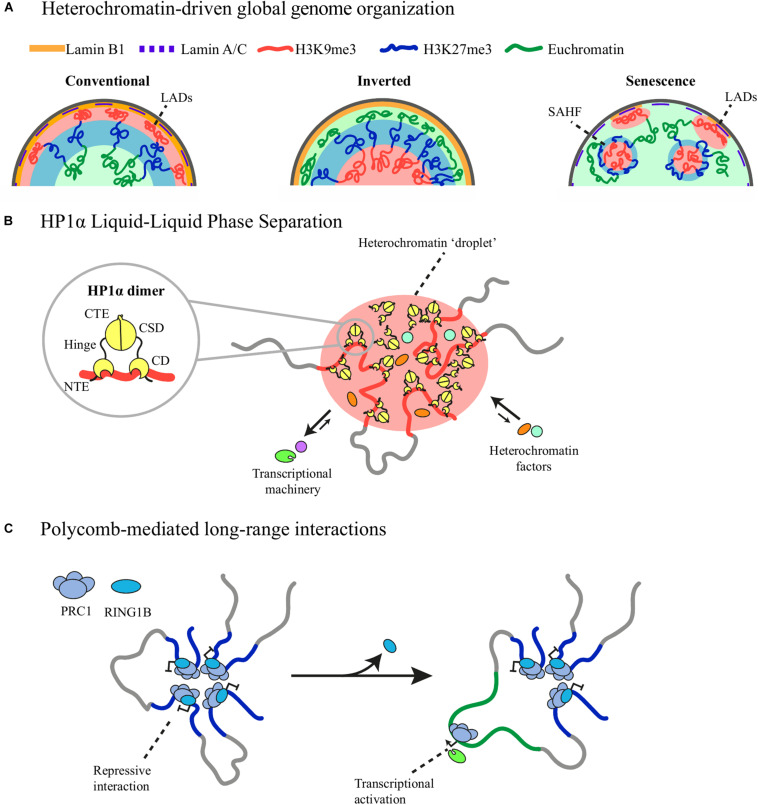
Distinct types of heterochromatin-driven genome organization. **(A)** Constitutive heterochromatin (red) tethering to the nuclear lamina forms LADs and instructs the conventional organization of the genome with euchromatin (green) located at the center of the nucleus adjacent to facultative heterochromatin (blue). Loss of heterochromatin tethering causes heterochromatin repositioning and inversion of the conventional organization in rod cells and the formation of SAHF in oncogene-induced senescence. **(B)** The chromodomain (CD) of HP1α recognizes and binds H3K9me3 histone mark. HP1α-bound heterochromatin comes together after local accumulation of HP1α promotes phase-separation mediated by its unstructured regions in the N-terminal (NTE), hinge and C-terminal (CTE) domains. Phase separation favors the exclusion of the transcriptional machinery from the heterochromatic phase and the inclusion of other heterochromatic factors. **(C)** RING1B, a member of PRC1, structures long-range interactions between promoters of Polycomb-repressed genes. Loss of RING1B causes loop disruption and concomitant gene activation.

Notably, heterochromatin sequestering to the nuclear periphery is also responsible for the conventional segregation pattern of euchromatin and heterochromatin. Rod photoreceptor cells of nocturnal mammals have an inverted nuclear architecture with euchromatin located at the periphery and heterochromatin at the center (Figure 1A; Solovei et al., 2009). In mouse rod cells, this unique chromatin distribution pattern is due to the downregulation of LBR expression around post-embryonic day 14 and absence of Lamin A/C expression (Solovei et al., 2009, 2013). Transgenic expression of LBR is sufficient to restore the conventional architecture in these cells, highlighting the importance of heterochromatin tethering as a large-scale organizing mechanism (Solovei et al., 2013). A similar segregation pattern is observed in human neutrophils where after Lamin B1 downregulation most of the accessible genome is located at the nuclear periphery, serving as focal points for global chromatin opening during NETosis (Chen et al., 2016). Heterochromatin segregation from euchromatin and its tethering to the nuclear lamina are therefore able to instruct global nuclear architecture.

A recent study explored the mechanisms underlying the inversion of heterochromatin positioning on mouse thymocytes before and after the deletion of LBR through Hi-C (Falk et al., 2019). Chromatin segregation can be observed in Hi-C interaction matrices since regions that share the same chromatin state tend to interact with each other frequently, forming distinguishable euchromatic and heterochromatic compartments (Lieberman-Aiden et al., 2009). Interestingly, relocating heterochromatin to the nuclear center after LBR depletion does not alter genome compartmentalization (Falk et al., 2019), hence the spatial segregation of euchromatin and heterochromatin probably depends on a higher affinity between regions with the same epigenetic marks rather than heterochromatin tethering to the nuclear periphery. A computational model that represents chromosomes as polymers of euchromatin, FH and CH, allowed measuring the effect of different affinities between chromatin states in the nuclear organization in order to reproduce the compartmentalization observed in Hi-C data and the inverted chromatin organization in LBR-null thymocyte microscopy (Falk et al., 2019). This model demonstrated that both the normal and the inverted architecture observed in LBR-null thymocytes are reproduced if CH regions exhibit a high affinity among themselves, whereas interactions between euchromatin regions are dispensable (Falk et al., 2019).

These findings show that heterochromatin-driven interactions are sufficient to determine global organization of the genome within the cell nucleus and alteration of heterochromatin positioning leads to dramatic reorganization, opening exciting new questions in the field as the exact mechanisms that mediate the highly frequent interactions between heterochromatin are largely unknown.

The nucleolar periphery constitutes a smaller heterochromatin compartment formed by NADs. NADs are enriched in satellite repeat clusters, inactive rDNA repeats, H3K9me3 repressed genes and some developmentally regulated genes rich in H3K27me3 (Vertii et al., 2019). Sequencing of DNA located around the nucleolus showed that some of these regions can alternate their location between NADs and LADs if one heterochromatic compartment is disrupted in order to maintain gene repression (van Koningsbruggen et al., 2010; Ragoczy et al., 2014), hence both heterochromatic compartments may be, to some extent, functionally redundant.

Heterochromatin positioning to the nucleolar periphery is mediated by nucleophosmins 1/2, as knockout of these proteins causes heterochromatin disruption and produces aberrant nucleolar morphology accompanied by transcriptional deregulation of ribosomal genes (Burns et al., 2003; Holmberg Olausson et al., 2014). Therefore, heterochromatin organization and stability not only assure proper gene regulation but assist genome large-scale organization and formation of subnuclear specialized compartments such as the nucleolus.

### Chromatin Compartments and Phase Separation

As stated previously, the spatial segregation of heterochromatin can be recovered in Hi-C data. Chromatin compartments were initially visualized in 1-Mb resolution Hi-C matrices as a characteristic plaid pattern of long-range interactions that reflect how euchromatic and heterochromatic regions interact with themselves forming two distinct genome-wide compartments (Lieberman-Aiden et al., 2009). Compartment A is enriched in actively transcribed genes, open chromatin and activating epigenetic marks like H3K36me3, H3K27ac and H3K4me3, whereas compartment B correlates with heterochromatic marks (Lieberman-Aiden et al., 2009). Identification of compartments in high-resolution heat maps showed that A/B compartments can be further subdivided into six smaller subcompartments each with particular chromatin modification signatures (Rao et al., 2014). Resolving the radial position of the genome by gradual chromatin digestion from the nuclear lamina toward the center coupled with sequencing, confirmed that euchromatic subcompartments are located more centrally than the H3K27me3 rich subcompartment, while the CH is retained at the nuclear periphery (Girelli et al., 2020). Thus, despite belonging to the same heterochromatic compartment, FH and CH can selectively mediate long-range chromatin interactions, reinforcing the idea that shared chromatin marks mediate chromatin segregation.

The molecular mechanisms responsible for chromatin compartmentalization have not been fully determined, however, recent evidence suggests that some chromatin components are able to induce Liquid-Liquid Phase Separation (LLPS) that results in the formation of supramolecular liquid droplets (i.e., chromatin compartments) immersed in a different, more diluted phase (i.e., the nucleoplasm), like oil droplets in water (Banani et al., 2017). Proteins with low complexity intrinsically disordered domains (LCDR) are able to form multivalent weak interactions among several partners that promote and stabilize LLPS (Erdel and Rippe, 2018).

HP1α is one of the best examples of a chromatin component able to phase-separate and drive LLPS of human, mouse and *Drosophila* heterochromatin (Larson et al., 2017; Strom et al., 2017). HP1α possesses unstructured regions in the N-terminal tail and the hinge domain can form liquid droplets *in vitro* and liquid-like droplets *in vivo* and can nucleate and fuse with other droplets as heterochromatin maturates, providing a novel mechanism of heterochromatin spreading (Strom et al., 2017). Moreover, HP1α droplets can selectively favor the inclusion of fluorescent-tagged HP1 interacting proteins into the heterochromatic phase while excluding others (Figure 1B; Larson et al., 2017), which raises the possibility that phase separation contributes to heterochromatin compartmentalization and stability. However, *in vivo* HP1α clusters do not show all the expected characteristics of liquid condensates as they do not have a round shape and are only partially susceptible to 1,6-hexanediol, an aliphatic alcohol that disrupts weak hydrophobic interactions (Strom et al., 2017), thus further studies are required to determine if these clusters are stabilized through LLPS or other phase separation mechanisms (Erdel et al., 2020).

Expression of an HP1α mutant that cannot be phosphorylated at the N-terminal tail or mutation of a lysine patch present in the hinge domain, reduce droplet formation (Larson et al., 2017). However, the effect of phase separation disturbance on chromatin compartmentalization has not been addressed. The identification of HP1α mutants that do not phase-separate may prove useful to study the role of heterochromatin LLPS in gene expression regulation, chromatin compartmentalization and heterochromatin assembly and stability.

Microscopy studies have proved that Polycomb-bound FH tends to aggregate in discrete foci, named Polycomb bodies (Cheutin and Cavalli, 2014). Interestingly, Chromobox 2 (CBX2), a member of the canonical PRC1 complex, has a LCDR domain that promotes phase separation *in vivo* and forms condensates with liquid-like properties (Tatavosian et al., 2019). Moreover, point mutations on the LCDR ablates Polycomb body assembly in NIH3T3 fibroblasts (Plys et al., 2019). Of note, other members of the CBX family are not able to phase-separate, which implies that the composition of PRC1 can regulate Polycomb body formation (Plys et al., 2019). In a different study, Polycomb bodies were disrupted after mutation of the sterile alpha motif of the PRC1 protein Polyhomeotic, which has not been shown to phase-separate (Wani et al., 2016). Thus formation of Polycomb bodies depends to a certain extent on LLPS.

Phase separation is a promising candidate to explain genome-wide compartmentalization as it has been shown to promote the condensation of chromatin regions with the same epigenetic marks, however, the principles of chromatin phase separation remain poorly understood as are the functional consequences of disturbing these phases. Whether heterochromatin LLPS is sufficient to induce global chromatin compartmentalization or whether it acts coordinately with other proteins or RNA belonging to the heterochromatin or euchromatin compartments is still unknown. In fact, there is evidence of LLPS properties in transcriptional factory assembly *in vivo* driven by interactions between the LCDR present in the CTD of RNA Pol II and transcription factors, though these interactions are short-lived (Chong et al., 2018).

Recent studies pointed out that genome compartmentalization can be regulated by cohesin, a protein involved in chromatin looping and TAD formation together with CTCF in mammalian cells (Haarhuis et al., 2017; Schwarzer et al., 2017). Hi-C experiments in mouse hepatocytes lacking the cohesin-loading factor Nipbl show an enhanced plaid pattern and compartmentalization and genome-wide loss of TAD structures (Schwarzer et al., 2017). Furthermore, cohesin removal in mESC enhances interactions between regions enriched in H3K27me3 and occupied by PcG proteins (Rhodes et al., 2020). In a study carried out in human HAP1 cells, increased cohesin association with DNA caused by knockout of the cohesin releasing factor WAPL, weakened genome compartments as noted by a fainted plaid pattern in Hi-C matrices and a decrease in far-*cis* interactions (Haarhuis et al., 2017). The observed strengthening of genome compartments after Nipbl depletion cannot be attributed to the loss of TAD organization since compartments remain unchanged after TAD loss caused by CTCF degradation in an auxin-inducible degron system in mESC (Nora et al., 2017). Cohesin antagonizes chromatin compartmentalization possibly restricting or altering the stability of heterochromatin-driven phase separation although the contribution of other mechanisms cannot be out ruled.

### Heterochromatin-Driven Chromatin Interactions

As previously stated, regions of Polycomb-bound heterochromatin can interact despite being located a significant linear distance apart to form PcG clusters. There are numerous examples of long-range loops between Polycomb-repressed regions that suggest Polycomb complexes can mediate chromatin interactions (Bantignies et al., 2011; Wani et al., 2016; Kundu et al., 2018). Recent evidence has revealed Polycomb-mediated long-range interactions between regions enriched in H3K27me3 that appear to be independent of cohesin and CTCF and finely regulated during development (Kundu et al., 2018; Du et al., 2020; Rhodes et al., 2020). Thus heterochromatin-driven genomic interactions, formed by possibly different mechanisms than the loop-extrusion model, act as important regulators of gene expression during development.

Promoter capture Hi-C of mESC showed a prominent network of long-range promoter-promoter interactions mediated by RING1B, a member of PRC1, with enriched interactions between the *Hox* gene clusters and genes encoding important developmental transcription factors rich in bivalent chromatin marks (Schoenfelder et al., 2015). RING1A/1B knockout abrogated this promoter network and caused gene expression upregulation, indicating that this global PRC1-dependent promoter network contributes to maintaining the silent state of developmentally regulated genes (Figure 1C; Schoenfelder et al., 2015). The strength of these interactions decreases during neuronal differentiation, as does RING1B occupancy, highlighting its implication regulating developmental processes (Bonev et al., 2017).

High-resolution Hi-C experiments in hematopoietic stem progenitor cells identified a group of long-range interactions between regions up to 117 Mb apart (Zhang et al., 2020). Conversely, the anchors of these interactions consist of regions with low levels of DNA methylation and are highly enriched in H3K27me3 with little or no detectable CTCF enrichment and are sensitive to H2K27me3 levels (Zhang et al., 2020), representing a class of interactions distinct from the ones mediated by CTCF and cohesin. These interactions are not present in differentiated cells and their disruption can alter the expression of nearby genes suggesting they may have a role in multipotency maintenance (Zhang et al., 2020).

Given the diversity of PcG proteins and their role in developmental processes, Polycomb-mediated interactions have emerged as topological regulators with major implications in cell-fate decisions. The mechanisms that underlie PcG mediated interactions however, are poorly understood. It will be interesting to evaluate the ability of other PcG proteins to mediate genomic interactions and/or phase-separate to expand our current knowledge on the mechanisms and functional importance of heterochromatin organization.

## Changes in Heterochromatin Organization in Aging and Disease

### Senescence-Associated Heterochromatin Foci

Oncogene-induced senescence (OIS) is accompanied by large-scale rearrangements of heterochromatin positioning forming nuclear structures known as Senescence-Associated Heterochromatin Foci (SAHF) (Narita et al., 2003). SAHF are heterochromatic domains with a distinctive organization consisting of a core of CH enriched in H3K9me3 and HP1 proteins encircled by a ring of FH rich in H3K27me3 (Figure 1A; Chandra et al., 2012). Other proteins shown to accumulate in SAHF are the histone variant macroH2A and the High-Mobility Group A (HMGA) proteins (Zhang et al., 2005; Narita et al., 2006). SAHF formation is largely diminished using shRNAs against HMGA1 or HMGA2, allowing cells to bypass senescence, thus SAHF are thought to aid cell-cycle arrest (Narita et al., 2006).

Notably, ChIP-seq experiments in growing and senescent cells showed that the distribution of H3K9me3 and H3K27me3 histone marks remain largely unchanged after OIS, thus SAFH formation reflects changes in the spatial positioning of pre-existing heterochromatin (Chandra et al., 2012). SAFH contain heterochromatic late-replicating regions with high A-T content that correspond to identified LADs (Chandra et al., 2015), consistent with the observed decrease in Lamin B1 levels in senescent cells (Shah et al., 2013). Moreover, a polymer model similar to the one used by Falk et al. (2019) predicts that SAHF establishment requires a high affinity among heterochromatic regions and a weak association between the nuclear lamina and chromatin (Chiang et al., 2019). Therefore SAFH form by heterochromatin-driven interactions between LADs detached from the nuclear periphery during senescence. Of note, ectopic expression of a dominant-negative form of HP1β unable to bind chromatin and that depletes all endogenous HP1 proteins from chromatin did not prevent SAFH formation (Zhang et al., 2007), hence the mechanisms driving SAFH assembly are probably different from HP1α-mediated phase separation.

Hi-C experiments conducted in growing and senescent cells showed that although TADs are conserved after senescence induction, the domains within SAHF lose insulation strength and interactions inside the TADs are reduced whereas interactions between distant heterochromatic regions are enhanced, thus the regions contained inside the SAHF experience local remodeling of their interactions landscapes (Chandra et al., 2015; Iwasaki et al., 2019). Whether these topological changes are related to changes in the levels of chromatin-bound CTCF or cohesin remains to be tested. Furthermore, a subset of genes activated upon OIS are located adjacent in the linear genome to regions that form SAHF and depend on SAHF formation to engage in TSS-TSS interactions that enhance their transcription (Sati et al., 2020), therefore heterochromatin repositioning during senescence causes upregulation of nearby genes by altering their interaction profiles.

OIS triggers extensive heterochromatin reorganization inside the nucleus and SAHF formation. Some cells are able to bypass senescence after SAHF disruption (Narita et al., 2006) and it has been suggested that SAHF ensure oncogene silencing and proper activation of senescence genes (Iwasaki et al., 2019; Sati et al., 2020) underscoring the importance of heterochromatin-mediated organization in the senescent phenotype.

### Heterochromatin Disorganization in Laminopathies

Laminopathies are a group of heterogeneous genetic diseases caused by mutations of the genes encoding nuclear lamins that cause lamin mislocalization, abnormal nuclear morphology and defects in chromatin organization which has led to the postulation of distinct non-mutually exclusive structural and transcriptional mechanisms responsible for laminopathies (Osmanagic-Myers and Foisner, 2019).

Heterochromatin detaches from the nuclear lamina in cells derived from patients with Hutchinson-Gilford Progeria Syndome (HGPS), a premature aging disorder caused by a mutation in *LMNA* that results in a form of Lamin A with an internal deletion of 50 amino acids (Eriksson et al., 2003). Furthermore, a decrease in heterochromatin marks is observed in HPGS cells before any detectable changes in the nuclear shape, which leads to transcriptional activation of normally repressed regions suggesting that HGPS cells fail to maintain heterochromatin identity and positioning contributing to the premature aging in HPGS patients (Shumaker et al., 2006; McCord et al., 2013).

Hi-C matrices of HGPS cells show a striking global loss of chromatin compartments and segregation, in agreement with the absence of heterochromatic clusters observed under the microscope (McCord et al., 2013). Interestingly the loss of chromatin compartmentalization cannot be explained solely by LAD detachment since both rod and OIS cells show chromatin compartments despite lacking heterochromatin tethering to the nuclear lamina (Chandra et al., 2015; Falk et al., 2019). Further investigation of the nuclear architecture of progeroid cells may unveil novel mechanisms driving chromatin segregation.

Chromatin segregation is also affected in *Drosophila* S2 cells after siRNA-mediated knockdown of the B-type lamin Dm0, which causes detachment and transcriptional activation of LADs (Ulianov et al., 2019). Expression of an N-terminally truncated version of Lamin C, the only A-type lamin in the fruit fly, or mutants modeled after the disease-causing forms of *LMNA* in humans resulted in alterations in nuclear morphology and muscle defects resembling the phenotype of muscular laminopathies (Schulze et al., 2009; Dialynas et al., 2010). This suggests that the pathological processes triggered after lamin loss and chromatin disorganization are conserved to some extent between *Drosophila* and mammals.

## Closing Remarks

Besides its role in gene expression silencing, heterochromatin plays an important role in 3D genome organization instructing the global positioning of the genome and the formation of chromatin compartments via strong interactions between heterochromatic regions and LLPS, though an extensive characterization of the factors able to induce and regulate heterochromatin LLPS is still needed. Heterochromatin factors also mediate long-range interactions independent of CTCF and cohesin, providing a mechanism of chromatin folding that regulates gene expression. Further work on the interplay between heterochromatin-driven organization and other known structural proteins may uncover new principles of genome organization that expand our current understanding of the forces driving chromatin segregation and structure.

## Author Contributions

AP-P and MF-M designed the reviewe’s content. AP-P wrote the manuscript with help, comments and editing by MF-M. All authors contributed to the article and approved the submitted version.

## Conflict of Interest

The authors declare that the research was conducted in the absence of any commercial or financial relationships that could be construed as a potential conflict of interest.
